# Tim-3 Relieves Experimental Autoimmune Encephalomyelitis by Suppressing MHC-II

**DOI:** 10.3389/fimmu.2021.770402

**Published:** 2022-01-13

**Authors:** Lili Tang, Ge Li, Yang Zheng, Chunmei Hou, Yang Gao, Ying Hao, Zhenfang Gao, Rongliang Mo, Yuxiang Li, Beifen Shen, Renxi Wang, Zhiding Wang, Gencheng Han

**Affiliations:** ^1^ Beijing Institute of Basic Medical Sciences, Beijing, China; ^2^ Department of Oncology, First Hospital, Jilin University, Changchun, China; ^3^ Department of Hematology and Oncology, International Cancer Center, Shenzhen Key Laboratory of Precision Medicine for Hematological Malignancies, Shenzhen University General Hospital, Shenzhen University Clinical Medical Academy, Shenzhen University Health Science Center, Shenzhen, China; ^4^ Beijing Institute of Brain Disorders, Laboratory of Brain Disorders, Ministry of Science and Technology, Collaborative Innovation Center for Brain Disorders, Capital Medical University, Beijing, China

**Keywords:** TIM-3, MHC-II, CIITA, EAE, antigen presentation, multiple sclerosis, T cell, macrophage

## Abstract

Tim-3, an immune checkpoint inhibitor, is widely expressed on the immune cells and contributes to immune tolerance. However, the mechanisms by which Tim-3 induces immune tolerance remain to be determined. Major histocompatibility complex II (MHC-II) plays a key role in antigen presentation and CD4^+^T cell activation. Dysregulated expressions of Tim-3 and MHC-II are associated with the pathogenesis of many autoimmune diseases including multiple sclerosis. Here we demonstrated that, by suppressing MHC-II expression in macrophages *via* the STAT1/CIITA pathway, Tim-3 inhibits MHC-II-mediated autoantigen presentation and CD4^+^T cell activation. As a result, overexpression or blockade of Tim-3 signaling in mice with experimental autoimmune encephalomyelitis (EAE) inhibited or increased MHC-II expression respectively and finally altered clinical outcomes. We thus identified a new mechanism by which Tim-3 induces immune tolerance *in vivo* and regulating the Tim-3-MHC-II signaling pathway is expected to provide a new solution for multiple sclerosis treatment.

## Introduction

T cell immunoglobulin mucin domain 3 (Tim-3) is widely expressed on the surface of many immune cells including T cells and macrophages (1, 2). As an immune checkpoint inhibitor, Tim-3 contributes to immune tolerance by inducing T cell apoptosis or by suppressing the activation of innate immune cells (1, 3). Deregulated upregulation of Tim-3 has been associated with many tumors or chronic infectious diseases, while dysregulated downregulation or dysfunction of Tim-3 leads to many kinds of autoimmune diseases such as multiple sclerosis (4) and rheumatoid arthritis (4–6). Recently, our findings and other reports showed that Tim-3 may control T cell response indirectly *via* regulating the function of innate immune cells (1, 3, 7). However, the mechanism by which Tim-3 mediates immune tolerance, especially innate immune tolerance, remains largely unclear.

MHC-II is expressed on many innate immune cells especially on professional antigen-presenting cells such as macrophages or dendritic cells. Following activation, MHC-II is upregulated and mediates antigen presentation and T cell activation. Unfortunately, dysregulated expression or variations of MHC-II lead to many immune disorders such as multiple sclerosis (8, 9). Investigations on the mechanisms by which MHC-II is regulated under different physiopathological conditions may provide useful solutions for many immune disorders. Multiple sclerosis (MS) is an autoimmune demyelinating and neurodegenerative disease of the central nervous system and the leading cause of non-traumatic neurological disability in young adults. Current therapeutic options for progressive multiple sclerosis remain comparatively disappointing and challenging.

The innate immune system is the first line of defense against antigens, and the adaptive immune system takes over by the innate immune system presentation of the antigens. Macrophages and microglia are important components of the innate immune system. They participate in the primary response to microorganisms and play a role in inflammatory responses, homeostasis, and tissue regeneration. In the initial phase of MS and EAE (an animal model of MS), macrophages from peripheral tissues infiltrate into the CNS and, together with residential microglia, contribute to the pathogenesis of MS. Strategies that target innate immune cells to prevent or treat immune disorders have shown therapeutical potential (10). Preventing multiple sclerosis by controlling the activity of macrophages/microglia may provide an alternative strategy.

Currently, it is found that the MHC-II molecule is the related gene for multiple sclerosis (11, 12). The expression of MHC-II is regulated by MHC class II transactivator (CIITA) (13). By reducing the MHC-II expression or blocking the MHC-II–peptide complex interaction on CD4^+^T cells, the multiple sclerosis progression can be significantly alleviated (14, 15). These data suggested that MHC-II may act as a therapeutic target for immune disorders such as multiple sclerosis. However, how MHC-II is regulated during different physiopathological conditions remains to be determined.

Here using cell-based, molecular biology and *in vivo* approaches, we identified a new way of MHC-II regulation; that is, by suppressing CIITA expression, Tim-3 inhibits MHC-II expression in macrophages and then downregulates the presentation of autoantigen MOG to CD4^+^T cells, finally leading to immune tolerance in mice with experimental autoimmune encephalomyelitis.

## Results

### Tim-3 Inhibits MHC-II Expression in Macrophages/Monocytes

**Figure 1:**
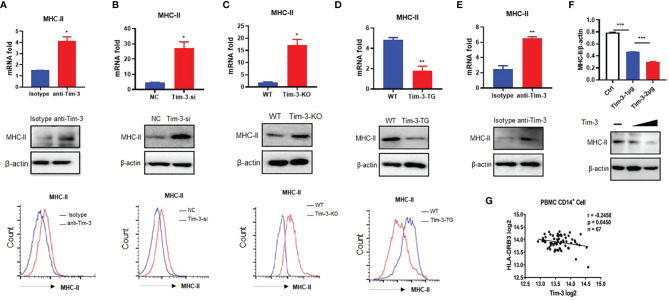
Tim-3 inhibits MHC-II expression in macrophages. **(A)** The MHC-II expression of RAW264.7 cells cocultured with anti-Tim-3 antibody (20 μg/ml) or isotype antibody for 24 h (upper, mean ± SD, n = 3). **(B)** Tim-3 knockdown (Tim-3-si) RAW264.7 cells and (negative control, NC) RAW264.7 cells (upper, mean ± SD, n = 3). **(C)** The peritoneal macrophages from Tim-3 knockout mice (Tim-3-KO) and WT mice (upper, mean ± SD, n = 6). **(D)** The peritoneal macrophages from Tim-3 transgenic (Tim-3-TG) mice and wild-type (WT) mice. The MHC-II expression was detected by cytometry, Western blot, and RT-PCR (mean ± SD, n = 6). **(E)** WT mice were treated with anti-Tim-3 antibody or isotype antibody (10 mg/kg) by intraperitoneal injection at 0 and 24 h, and peritoneal macrophages were collected at 48 h to detect MHC-II protein and gene levels (mean ± SD, n = 6). **(F)** 1 and 2 μg of Tim-3 plasmid and 2 μg of vector plasmid were transferred into HEK-293T cells, respectively, and MHC-II protein level was detected at 48 h. **(G)** The expression data of Tim-3 and MHC-II in CD14+ monocytes/macrophages from 67 healthy individuals were collected from the GEO dataset (GSE34151), and their correlation was analyzed (*p < 0.05; **p < 0.01; ***p < 0.001).

In our previous studies, we demonstrated that Tim-3 inhibits the activity of the phagocytosis (16) and MHC-I antigen presentation (7) of macrophages. There is great interest to keep on investigating whether Tim-3 inhibits other antigen presentation functions in macrophages and other immune functions. MHC-II expression was increased by anti-Tim-3 antibody inhibition in mouse macrophage cell line RAW264.7 (**Figure 1A**). Tim-3 was also knocked down by siRNA in RAW264.7, whose results (**Figure 1B**) showed that MHC-II expression was increased by Tim-3 knockdown. To test this further, the peritoneal macrophages of wild-type C57BL/6 mice (WT) mice, Tim-3-KO, and Tim-3-TG mice were extracted and analyzed. The MHC-II expression in Tim-3-KO mice was increased in macrophages compared with WT mice (**Figure 1C**), and MHC-II expression was decreased in macrophages from Tim-3-TG mice compared with WT mice (**Figure 1D**). WT mice were intraperitoneally injected with anti-Tim-3 antibody or control antibody, and peritoneal macrophages were harvested after 48 h. The MHC-II expression was increased after anti-Tim-3 antibody inhibition (**Figure 1E**). In addition, the expression of MHC-II on Tim-3 transgenic HEK-293T cells was decreased at both the mRNA and protein levels (**Figure 1F**). To find out whether Tim-3 also inhibited MHC-II expression on human monocytes/macrophages, Tim-3 and HLA-DR mRNA expression data from the GEO dataset (GSE34151) (7, 17) were collected. The 67 healthy individual samples were analyzed and showed an inverse correlation (r = -0.2458, p = 0.0450) between the expression of Tim-3 and MHC-II (HLA-DR) in CD14+ monocytes/macrophages (**Figure 1G**). These results suggest that Tim-3 signaling inhibited MHC-II expression not only in mouse macrophages but also in human macrophages.

### Tim-3 Inhibits CIITA Expression in Macrophages

**Figure 2:**
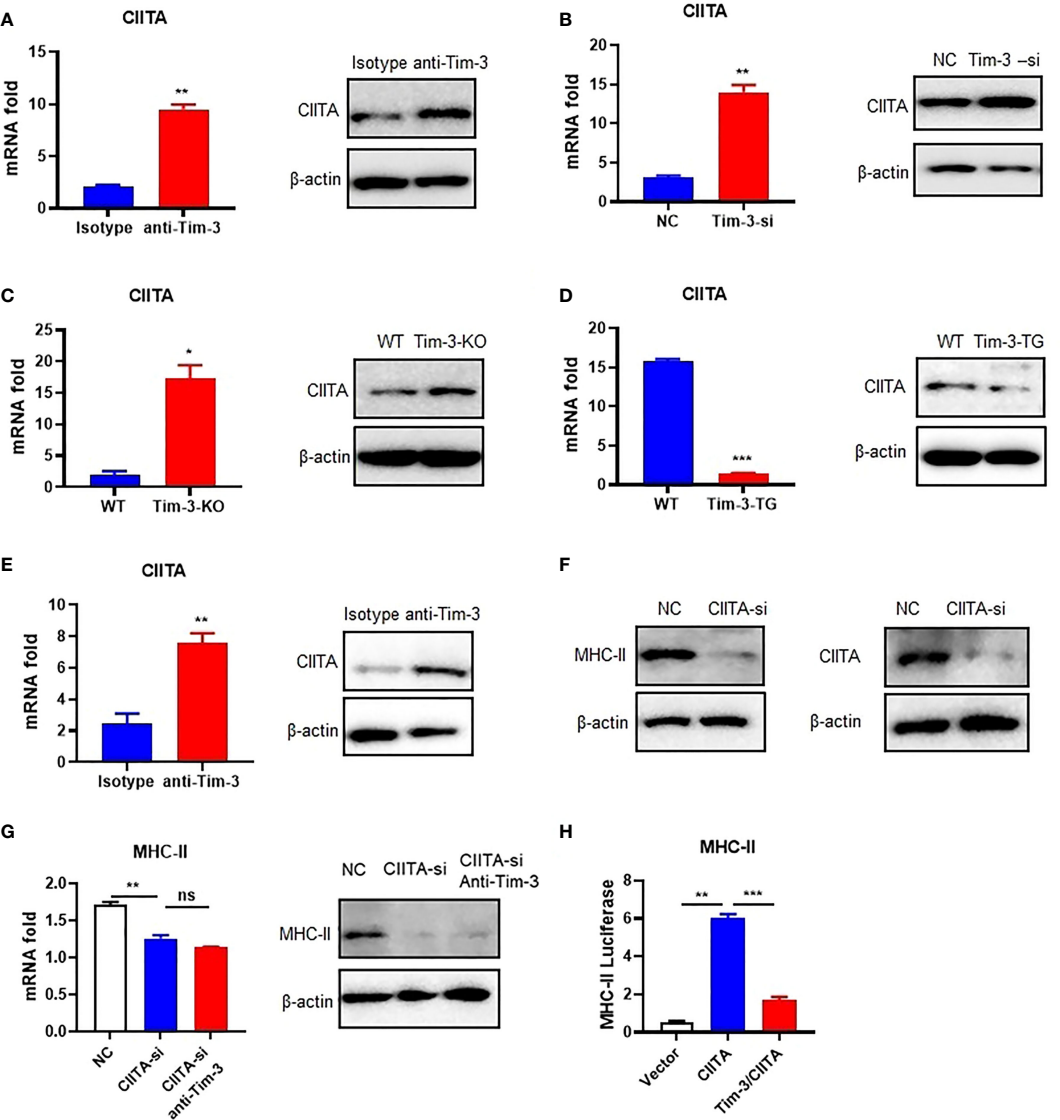
Tim-3 signaling inhibits MHC-II expression through CIITA in macrophages. **(A)** The CIITA expression of RAW264.7 cells cocultured with anti-Tim-3 antibody (20 μg/ml) or isotype antibody for 24 h (left, mean ± SD, n = 3). **(B)** Tim-3-si RAW264.7 cells and RAW264.7 cells (left, mean ± SD, n = 3). **(C)** The peritoneal macrophages from Tim-3-KO and WT mice. The CIITA expression was detected by Western blot and RT-PCR (mean ± SD, n = 6). **(D)** The peritoneal macrophages from Tim-3-TG mice and WT mice (left, mean ± SD, n = 6). **(E)** WT mice were treated with anti-Tim-3 antibody or isotype antibody (10 mg/kg) by intraperitoneal injection at 0 and 24 h, and peritoneal macrophages were collected at 48 h to detect CIITA protein and gene levels (mean ± SD, n = 6). **(F)** siRNA-CIITA was transfection with RAW264.7 cells; CIITA and MHC-II expression was detected by Western blot at 48 h. **(G)** siRNA-CIITA was transfection with RAW264.7 cells cocultured with or without anti-Tim-3 antibody, and MHC-II expression was detected by RT-PCR (mean ± SD, n = 3) and Western blot at 48 h. **(H)** HEK-293T cells were transferred into pGL3-MHC-II reporter plasmid and CIITA plasmid, with or without Tim-3 plasmid for 48 h. Dual-fluorescence was analyzed (mean ± SD, n = 3). (*p < 0.05; **p < 0.01; ***p < 0.001). ns, Not Significant.

Tim-3 signaling inhibited MHC-II expression, but how Tim-3 inhibits MHC-II is still unclear. CIITA is the transcription factor of MHC-II (13), and Tim-3 may also inhibit the CIITA expression. To test the hypothesis, the Tim-3 function to CIITA was explored. CIITA expression was increased by anti-Tim-3 antibody inhibition in RAW264.7 (**Figure 2A**). Tim-3 was also knocked down by siRNA, whose results (**Figure 2B**) showed that CIITA expression was increased. To test this further, the peritoneal macrophages of WT, Tim-3-KO, and Tim-3-TG mice were extracted and analyzed. The CIITA expression in Tim-3-KO mice was increased in macrophages compared with WT mice (**Figure 2C**), and CIITA expression was decreased in macrophages from Tim-3-TG mice compared with WT mice (**Figure 2D**). The CIITA expression was increased after anti-Tim-3 antibody inhibition in mice (**Figure 2E**). These results suggest that Tim-3 signaling inhibited CIITA expression in macrophages.

### Tim-3 Signaling Inhibits MHC-II Expression Through CIITA in Macrophages

After determining the inhibitory effects of Tim-3 on CIITA, we examined whether Tim-3 inhibits MHC-II expression through CIITA. To test this hypothesis, we silenced CIITA in RAW264.7 cells by siRNA (**Figure 2F**). Knockdown of CIITA led to decreased MHC-II mRNA and protein expression, which cannot be reversed by Tim-3 antibody blockage in RAW264.7 (**Figure 2G**). In addition, the dual-luciferase reporter assay revealed that CIITA-induced upregulation of MHC-II in HEK-293T cells could be reversed when Tim-3 was co-transfected (**Figure 2H**). These results showed that Tim-3 signaling inhibits MHC-II expression in macrophages through CIITA.

### STAT1 Acts as a Signaling Adaptor for Tim-3-Mediated Suppression on the CIITA-MHC-II Pathway

**Figure 3:**
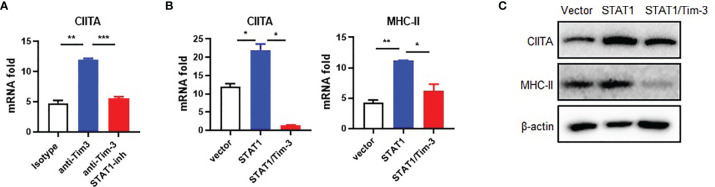
Tim-3 signaling inhibits CIITA expression through STAT1 in macrophages. **(A)** RAW264.7 cells were incubated with 20 μg/ml anti-Tim-3 antibody or control antibody for 24 h in the presence of the STAT1 inhibitor fludarabine (10 μM) or dimethyl sulfoxide control and then were collected, and CIITA mRNA levels were examined by RT-PCR (mean ± SD, n = 3). **(B)** HEK-293T cells were transfected with STAT1 plasmid, with or without Tim-3 plasmid for 48 h, and the mRNA levels of CIITA (mean ± SD, n = 3) and MHC-II (mean ± SD, n = 3) were detected. **(C)** HEK-293T cells were transfected with STAT1 plasmid, with or without Tim-3 plasmid and lysed, and the protein levels of CIITA and MHC-II were analyzed at 48 h (*p < 0.05; **p < 0.01; ***p < 0.001).

We previously found that STAT1 helps Tim-3 to transduce signal (7). The STAT1-CIITA signaling pathway is involved in the regulation of MHC-II transcription (18–20). To test whether Tim-3 inhibits CIITA expression *via* STAT1, we first examined the Tim-3 and STAT1 signal to CIITA. The results showed that the expression of CIITA mRNA in RAW264.7 cells increased after Tim-3 antibody blockage, which could be reversed by the STAT1 inhibitor fludarabine (**Figure 3A**). CIITA and MHC-II mRNA increased after STAT1 transgenetic overexpression and reversed by Tim-3 co-overexpression (**Figure 3B**). Furthermore, the CIITA and MHC-II expression in protein level can be reversed by Tim-3 co-overexpression (**Figure 3C**). These results showed that Tim-3 signaling inhibits MHC-II expression in macrophages through STAT1-CIITA.

### Tim-3 Inhibits MHC-II and Ameliorates the EAE Mouse Model

**Figure 4:**
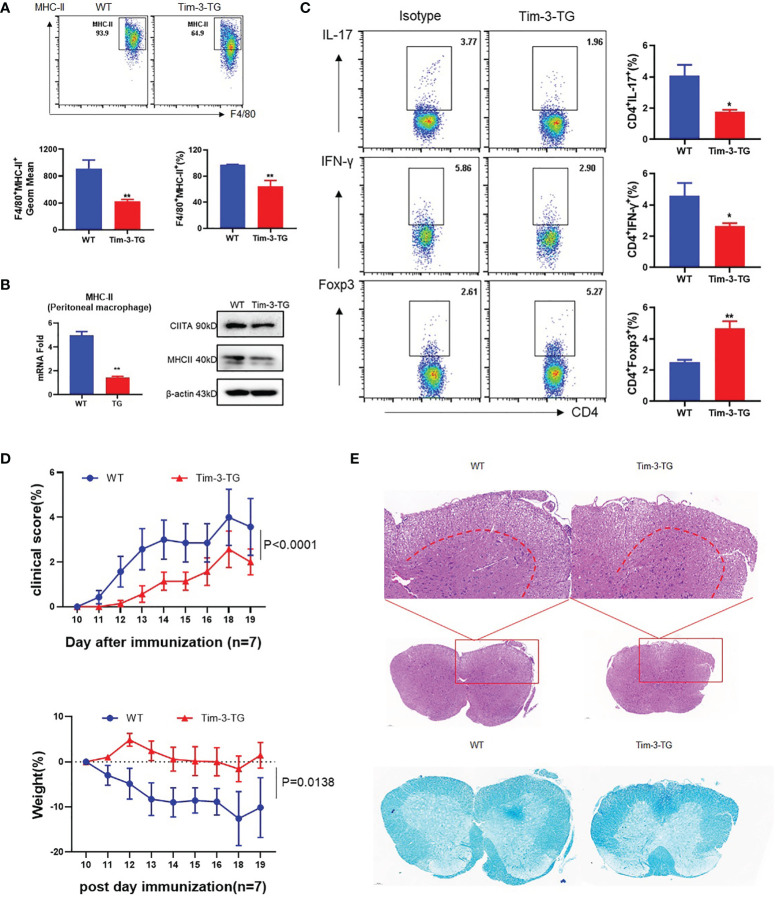
Tim-3 inhibits MHC-II and ameliorates the EAE mouse model. MOG was emulsified into CFA, WT, and Tim-3-TG mice (n = 8) were immunized subcutaneously, and pertussis toxin (500 ng/mouse) was injected intraperitoneally at 2 and 48 h after immunization. On the 20th day after immunization, the mouse spinal cords, peritoneal macrophages, and spleens were harvested. **(A)** Flow cytometry was used to detect the expression of MHC-II on the surface of macrophages. The percentage of MHC-II and the GeoMean were counted (mean ± SD, n = 6–8), **(B)** and the expression level of MHC-II was detected by RT-PCR (mean ± SD, n = 6) and Western blotting. **(C)** Flow cytometry was used to detect the activation levels of IL-17^+^CD4^+^T, IFN-γ^+^CD4^+^T, and Foxp3^+^CD4^+^Tcells in splenic lymphocytes (mean ± SD, n = 8–10). **(D)** The weight and clinical scores of the EAE mice were recorded from the 10th day. **(E)** The mouse spinal cord tissue was isolated and stained with HE and LFB (*p < 0.05; **p < 0.01).

To study whether Tim-3 inhibits the macrophage MHC-II expression and antigen presentation *in vivo*, the EAE model was constructed on wild-type (WT) or Tim-3-TG C57BL/6 mice. The dynamic development and progression of EAE were monitored for the disease scores and body weights. MHC-II and CIITA expression was decreased in EAE mice macrophages from Tim-3-TG mice compared with WT mice (**Figures 4A, B**). CD4+ T cells in the Tim-3-TG group differentiate fewer IFN-γ+CD4+ T (Th1) cells and IL17+CD4+ T (Th17) cells, with more Foxp3+CD4+ T (Treg) cells (**Figure 4C**). Tim-3-TG significantly reduced the development and severity of EAE by disease score and weight loss, with less inflammatory cell infiltration and demyelination lesions in white matter by H&E and LFB staining (**Figures 4D, E**). These data indicated Tim-3 can attenuate the development and progression of EAE in mice.

**Figure 5:**
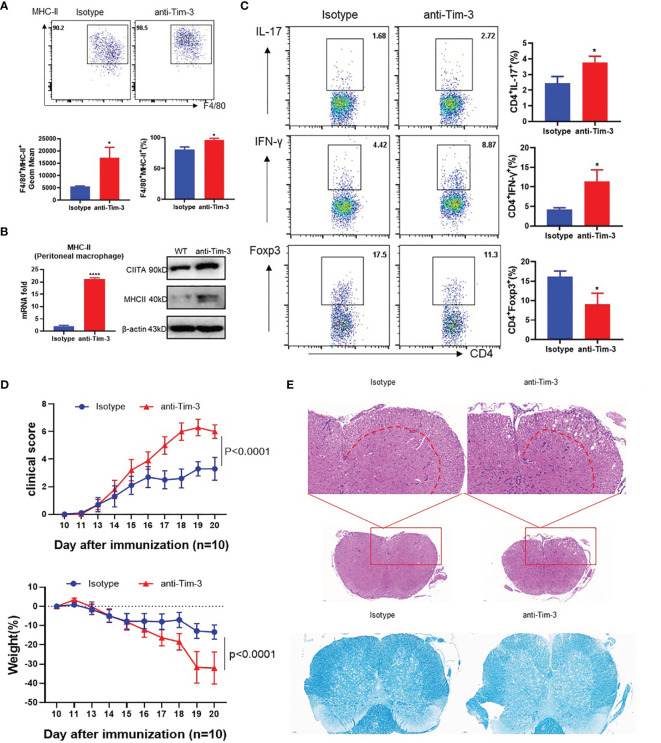
Blockade of Tim-3 exacerbated multiple sclerosis in the EAE model. MOG was emulsified into CFA, mice were immunized subcutaneously, and pertussis toxin (500 ng/mouse) was injected intraperitoneally at 2 and 48 h after immunization. The immunized WT mice were divided into two groups (n = 10): one group is injected with anti-Tim-3 antibody, and the other group is injected with isotype antibody (10 mg/kg, intraperitoneal injection every other day. On the 20th day after immunization, the mouse spinal cords, peritoneal macrophages, and spleens were harvested. **(A)** Flow cytometry was used to detect the expression of MHC-II on the surface of macrophages. The percentage of MHC-II and the GeoMean were counted (mean ± SD, n = 5), **(B)** and the expression level of MHC-II was detected by RT-PCR (mean ± SD, n = 6) and Western blotting. **(C)** Flow cytometry was used to detect the activation levels of IL-17^+^CD4^+^T, IFN-γ^+^CD4^+^T, and Foxp3^+^CD4^+^T cells in splenic lymphocytes (mean ± SD, n = 6–10). **(D)** The weight and clinical scores of the EAE mice were recorded from the 10th day. **(E)** The mouse spinal cord tissue was isolated and stained with HE and LFB (*p < 0.05; ****p < 0.0001).

CIITA and MHC-II expression in peritoneal macrophages of EAE mice was upregulated in the Tim-3 blockage group (**Figures 5A, B**). CD4+ T cells in the anti-Tim-3 group differentiate more IFN-γ+CD4+ T (Th1) cells and IL17+CD4+ T (Th17) cells, with few Foxp3+CD4+ T (Treg) cells (**Figure 5C**). After Tim-3 signaling blockage, the severity of EAE is significantly developed by disease score and weight loss (**Figure 5D**). H&E and LFB staining showed that inflammatory cell infiltration and demyelination lesions in white matter were significantly increased in the Tim-3 blockage group (**Figure 5E**). The data indicated that Tim-3 blockage exacerbated the development and progression of EAE in mice.

These data indicate that Tim-3 could inhibit MHC-II expression, which decreased macrophage antigen presentation and stimulation to CD4+ T cells with increased anti-inflammatory Treg and decreased pro-inflammatory Th1 and Th17 CD4+ T cells. Tim-3 can relieve EAE mouse spinal cord demyelination and improve clinical scores by inhibiting MHC-II expression and CD4+ T cell stimulation.

**Figure 6:**
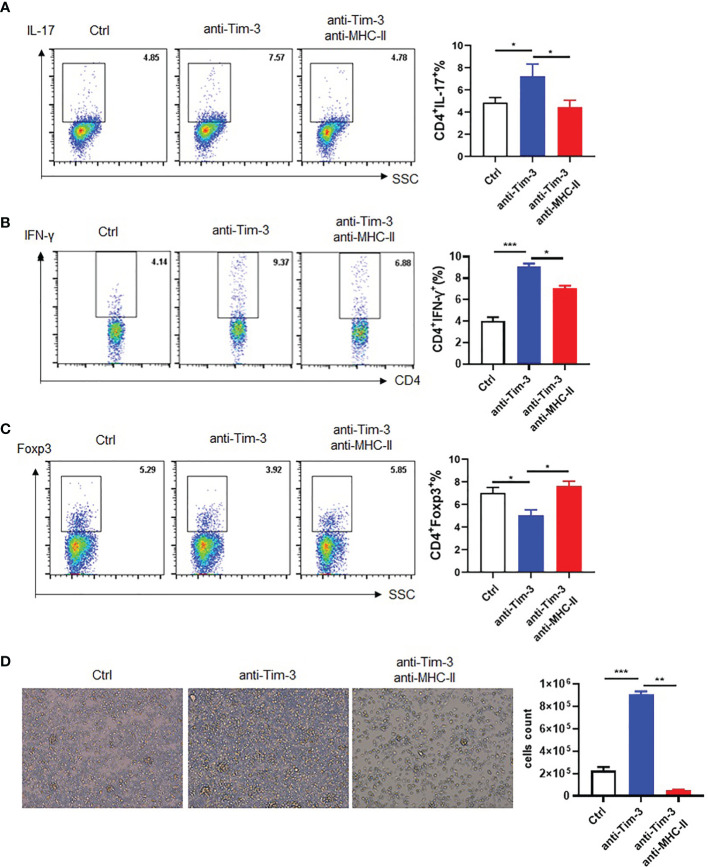
Tim-3 inhibits macrophage MHC-II antigen presentation *in vitro*. Peritoneal macrophages were collected from WT mice, and anti-Tim-3 antibody (10 μg/ml) was added to culture overnight then incubated with MOG (10 μg/ml) for 4 h. Moreover, they were incubated with or without anti-MHC-II antibody (10 μg/ml) for 30 min, and then splenic lymphocytes were added to coculture for 24 h. The splenic lymphocyte suspension was collected to detect the ratio of **(A)** IL-17^+^CD4^+^T (mean ± SD, n = 4), **(B)** IFN-γ^+^CD4^+^T (mean ± SD, n = 4), and **(C)** Foxp3^+^CD4^+^T cells by flow cytometry (mean ± SD, n = 3). **(D)** Splenic lymphocytes of WT mice were cocultured for 3 days and photographed with a microscope, and then spleen cells were for collected for counting (mean ± SD, n = 3) (*p < 0.05; ***p < 0.001).

MHC-II plays a critical role in antigen presentation and CD4+ T cell activation. To verify if Tim-3 regulates the MHC-II antigen presentation function and control, anti-Tim-3 antibody was added to peritoneal macrophages and incubated with the MOG35-55 peptide for MHC-II presentation. Then, mouse spleen cells were added to the macrophages with or without anti-MHC-II antibody blockage. The result is analyzed by FACS, which showed that Tim-3 signal blockage could decrease anti-inflammatory Treg (**Figure 6C**) and increased pro-inflammatory Th1 (**Figure 6B**) and Th17 CD4+ T (**Figure 6A**) cells through MHC-II presentation. The spleen cells were also had more proliferation by inhibiting the Tim-3 signal through MHC-II presentation (**Figure 6D**). The results showed that Tim-3 can inhibit macrophage MHC-II antigen presentation to CD4+ T cells *in vitro*.

## Discussion

In this study, we found that Tim-3 inhibits MHC-II expression in macrophages *via* the STAT1/CIITA pathway and inhibits antigen presentation and CD4+T cell activation in the EAE model. Moreover, blockade of Tim-3 signaling in EAE mice increased MHC-II expression and altered clinical outcomes. We identified a new mechanism that Tim-3 induces immune tolerance by MHC-II presentation, which paves a new way for multiple sclerosis treatment and other MHC-II related diseases.

In our previous studies, Tim-3 inhibits the MHC-I expression and antigen presentation function. Wang et al. (7) found that MHC-I-restricted antigen presentation by macrophages was inhibited by Tim-3-NLRC5 both *in vitro* and in a Listeria monocytogenes infection model *in vivo*. Li et al. (21) also found that Tim-3 blockage increases the expression of MHC-I on macrophages and promotes the activation of VSV-specific CD8+ T cells and also markedly attenuates vesicular stomatitis virus (VSV) encephalitis by decreased mortality and improved neuroethology in mice. In this study, we found that Tim-3 can inhibit the MHC-II expression and antigen presentation to CD4+ T cells, which relieves EAE mouse spinal cord demyelination and improves clinical scores. Tim-3 could also shift CD4+ T cells from a pro­inflammatory Th1 and Th17 phenotype to a less damaging, anti­inflammatory Treg phenotype. These studies showed that Tim-3 inhibits not only MHC-I expression and function but also MHC-II in different animal models, which suggests that Tim-3 may have more function and potential in macrophage antigen function.

Understanding MHC-II antigen presentation in health and disease may be critical for developing tools to control autoimmune responses and to modulate strong responses against infections and cancer (8). CD4+ T cells that interact with MHC-II–bound peptides then promote B cell differentiation and antibody production, as well as CD8+ T cell responses. The immune process of MHC-II is vital and tightly controlled, with one step of unregulated responses that can promote infectious diseases, autoimmune diseases, and cancer. MHC-II alleles are indeed associated with autoimmune diseases and are often the strongest risk factors (22), such as MS, type 1 diabetes, systemic lupus erythematosus, ulcerative colitis, Crohn’s disease, and rheumatoid arthritis. In this study, we found that Tim-3 inhibits MHC-II expression and antigen presentation with EAE model amelioration, which indicates that the Tim-3-targeting therapeutic strategy could have more potential for cancers and infectious and autoimmune diseases. There are two possible solutions to the design of a Tim-3-targeting therapy (2). One is IgG4 monoclonal antibodies, which could inhibit Tim-3 signaling in the immune system. The other is the use of IgG1 Tim-3 antibodies with CDC and ADCC function, which could kill dysfunctional immune cells with Tim-3 expression and differentiated monocytes. Elimination of dysfunctional Tim-3-expressing immune cells could be a new way to address immune tolerance (2). These problems are still waiting to be solved.

Multiple sclerosis (MS) is a chronic inflammatory disease of the central nervous system that involves demyelination and axonal degeneration. The multiple sclerosis treatment began with the approval of IFN-β and glatiramer acetate, then the first monoclonal antibody natalizumab, followed by oral medications (fingolimod, teriflunomide, dimethyl fumarate, and cladribine). Recently, new monoclonal antibodies (alemtuzumab and ocrelizumab) have been approved (23). MS treatment remains some problems. One is that progressive MS degenerative mechanisms differ from RRMS inflammatory mechanisms and that the neurodegenerative processes are not resolved by immunomodulatory compounds (24). Another is that animal models limit the understanding of pathophysiological mechanisms in progressive MS (25, 26). The current understanding is that progressive MS is characterized by chronic inflammation behind a closed blood–brain barrier with activation of microglia and continued involvement of T cells and B cells (24).

The goal of therapeutic modulation of T cell composition and differentiation in progressive MS is generally to normalize inflammation and minimize any involvement of T cell infiltrates. T cells can be found in the brain and spinal cord parenchyma of patients with PPMS and RRMS (27). Targeting T cells to slow disease progression needs more research. However, several interesting approaches could effectively target T cells, some of which are under investigation.

**Figure 7:**
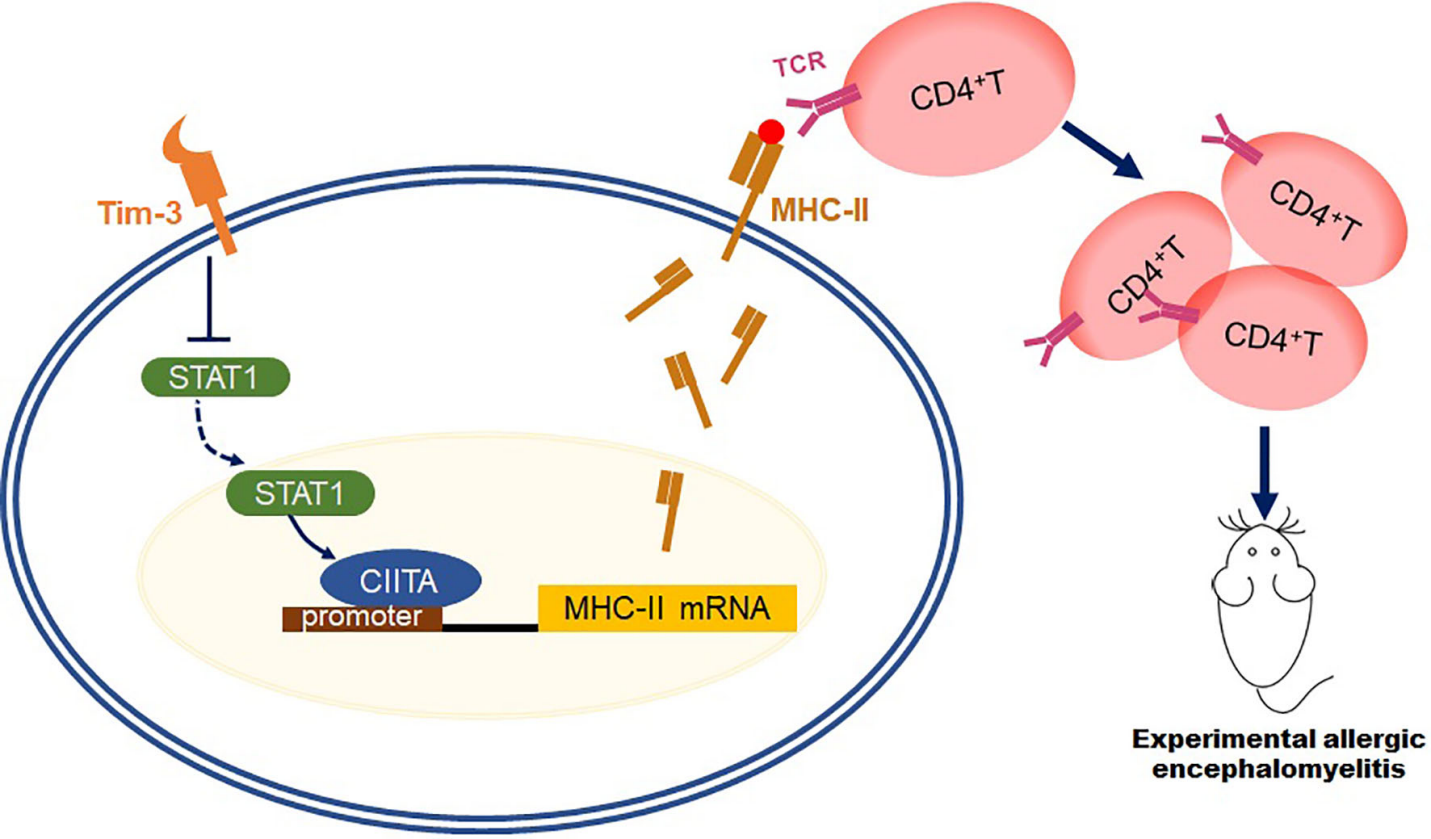
Schematic diagram of how Tim-3 inhibits MHC-II expression and relieves the EAE model. This schematic diagram demonstrated that Tim-3 suppresses MHC-II expression in macrophages *via* the STAT1/CIITA pathway, and Tim-3 inhibits MHC-II-mediated autoantigen presentation and CD4+T cell activation, which facilitated experimental autoimmune encephalomyelitis in mice.

In summary, this study aims at finding the mechanism that Tim-3 could inhibit MHC-II expression and antigen presentation function through STAT1-CIITA, which relieves the EAE model (**Figure 7**), paving the way for Tim-3 as a potential therapeutic target to clinical usage.

## Materials and Methods

### Animals

Female 6–8-week-old C57BL/6J mice were purchased from Sibefu; Tim-3 transgenic mice were obtained from Cyagen Biosciences (Guangzhou, China). All mice were raised in a specific pathogen-free condition with free access to food and water. All experiments were performed according to the protocol approved by the Animal Ethics Committee of the Academy of Military Sciences (permit number: 2020-715).

### EAE Induction

EAE was established as described in the literature (28, 29). Briefly, the complete Freund’s adjuvant (CFA, Sigma, F5881) was added with 50 mg of Mycobacterium tuberculosis (H37Ra, BD, 0052519) to prepare a final concentration of 6 mg/ml. MOG_35-55_ (SBS Genetech, Beijing) was emulsified with CFA (0.3 mg MOG_35-55_ and 0.6 mg H37Ra per mouse). The mice were immunized subcutaneously on the back, and Pertussis toxin (PTX, List Biological Laboratories, 180243A1) was injected intraperitoneally at 0 and 48 h after immunization (500 ng PTX per mouse each time). Groping, twenty C57BL/6J mice were immunized and randomly divided into two groups: anti-Tim-3 antibody (21) group and control antibody group (10 mg/kg, i.p, every other day). Eight Tim-3-TG mice and eight WT mice were immunized. The weight and clinical score of the EAE were recorded from the 10th day after immunization (30, 31).

### H&E and LFB Staining

The mice were sacrificed on the 20th day after immunization. The mouse spinal cord was obtained and fixed with 4% paraformaldehyde, then paraffin embedding and tissue sectioning were performed. Perform hematoxylin and eosin staining was done to assess the state of the cortical and neuronal cells of the parenchymal. The Luxol fast blue method is used to stain myelin integrity. The microscope image was scanned with Pannoramic DESK, P-MIDI, P250 (Hungary, 3DHISTECH), and analyzed with Pannoramic Scanner software.

### Cell Culture and Transfection

RAW264.7 cells and HEK-293T cells were from ATCC and were cultured under the culture guidelines. si-RAW264.7 cells (stably knocked out Tim-3) come from our laboratory (16). All cells using Dulbecco’s modified Eagle’s medium supplemented with 10% fetal bovine serum, streptomycin (50 μg/ml), and erythromycin (5 μg/ml) were cultured in an incubator (37°C, 5% CO2).

For cell transfection, Tim-3 plasmid gradient transfection was as follows: Vector or 1- and 2-μg Tim-3 plasmids were transfected into HEK-293T cells, respectively. Tim-3, CIITA, and STAT1 plasmids were transfected into HEK-293T cells alone or in combination and cultured for 48 h after transfection. The plasmid was previously stored in our laboratory. The CIITA knockdown, Vector, and CIITA siRNA (Guangzhou Ribobio, S1105) were transfected into RAW264.7 cells for 48 h. In the cell blocking experiment, RAW264.7 cells were cocultured with anti-Tim-3 antibody (20 μg/ml) or isotype antibody for 24 h. Also, 10 μM fludarabine (STAT1 inhibitor) was added on RAW264.7 cells which were then cocultured with anti-Tim-3 antibody (20 μg/ml) for 24 h.

### Western Blotting

The cells were collected and lysed with the lysis buffer, in which protease and phosphatase inhibitors were added, then the supernatant was centrifuged and collected. The loading buffer was added and boiled for 10 min and then stored at -20°C. The protein concentrations of the sample were assessed using BCA Protein Analysis Kit (Thermo Fisher, TF268083). The protein sample was boiled for 5 min and was electrophoresed by SDS-PAGE (sodium dodecyl sulfate-polyacrylamide gel electrophoresis) and then transferred to a polyvinylidene difluoride (PVDF) membrane. Then, the membrane was blocked for 1 h with TBST containing 5% milk at room temperature. The blots were incubated with the following primary antibodies overnight at 4°C: anti-CIITA antibody (Abcam, ab49132), anti-MHC-II antibody (Abcam, ab180779), and anti-mouse β-actin antibody (Abcam, ab8227). The dilution method of the above antibodies is according to the instructions. Afterward, the membrane was washed three times with TBST, 10 min each time, then incubated with Goat anti-rabbit IgG or Goat anti-mouse IgG antibodies for 40 min at room temperature, then washed three times with TBST for 10 min each time.

### Real-Time PCR

The peritoneal macrophages of Tim-3-TG and WT EAE mice were collected, and RNA was extracted according to the TRIzol instructions (Ambion, 28218) (32). The concentration and purity of RNA were assessed by a Q5000 ultraviolet spectrophotometer (Quawell Technology Inc., Sunnyvale, USA). The RNA was reverse-transcribed by using TransScript First-Strand cDNA Synthesis SuperMix (TransGen Biotech, N20730). Quantitative real-time PCR was performed using UltraSYBR One-Step RT-qPCR Kit (Beijing ComWin Biotech, CW0659) and a LightCycler 480 PCR system. The relative expression of interesting genes was detected using the 2^-ΔΔCt^ method, and internal control (18S ribosomal mRNA) was used for normalization of the target genes (7). The interesting primer sequences were shown as follows:

18S: sense 5′-TTGACGGAAGGGCACCACCAG-3′
Anti-sense 5′-GCACCACCACCACGGAATCG-3′
MHC-II: sense 5′-GCGGAGAGTTGAGCCTACG-3′
Anti-sense 5′-CCAGGAGGTTGTGGTGTTCC-3′
CIITA: sense 5′-TGTTTTGGATGCTGCAAGGC-3′
Anti-sense 5′-AAGGCACAGTGGTATTCCCG-3′

### Dual-Luciferase Reporter Assay

The pGL3-MHC-II (1 μg) and pGL3-CIITA (1 μg) plasmids (Shenggong, Beijing, China) were transferred into HEK-293T cells, with or without Tim-3 plasmid, and cultured for 48 h. The luminescence was detected with the Dual-Luciferase Report Assay System (Promega, E1910). Briefly, the cells were washed with PBS once and 200 μl lysis buffer was added, then 20 μl was added to a 96-well plate, 50 μl 1× Gold’s reagent was added to each well, and firefly luciferin was measured immediately, and then 50 μl 1× Stop reagent was added to measure Renilla luciferase. Finally, the relative of firefly fluorescein/renilla fluorescein was calculated (33).

### FACS Analysis

The spleen lymphocytes of EAE mice (on the 20th day after immunization) were separated with Ficoll density gradient centrifugation. Peritoneal macrophages were isolated as previously described (7), washed twice with FACS (containing 2% fetal bovine serum), then stained with APC anti-mouse F4/80 antibody (BioLegend, 123116) and PE anti-mouse MHC-II antibody (BioLegend, B117132), incubated at 37°C for 30 min, and then washed once with FASC and resuspended in 1% paraformaldehyde. For staining of spleen cells, firstly, the cells were incubated with cell stimulation cocktail plus protein transport inhibitors (Invitrogen, 2260623) for 4–6 h, washed with FACS once for staining with PerCP anti-mouse CD4 antibody (BioLegend, 100538), and then washed with FACS; 200 μl fixation/permer buffer (Invitrogen, 2220750) was added at 4°C overnight, and then permer buffer dilution washing and staining were done on the APC anti-mouse IFN-γ antibody (BioLegend, 505810), PE anti-mouse IL-17 antibody (BioLegend, 506904), and APC anti-mouse Foxp3 antibody (eBioscience, E07303-1635), and finally washing with permer buffer and resuspension were performed.

### MHC-II Neutralization Experiment *In Vitro*

Peritoneal macrophages and spleen cells of wild-type mice were obtained according to the above method. Then, anti-Tim-3 antibody was added to the peritoneal macrophages to incubate overnight, then MOG_35-55_ was also added to incubate for 4 h, and then anti-MHC-II antibody (Invitrogen, 2190425) was added to incubate for 30 min, and splenic lymphocytes were finally added and, after 24 h, photographed with a microscope; then, spleen cells for were collected for counting and then stained with PerCP anti-mouse CD4 antibody and APC anti-mouse IFN-γ antibody.

### Statistical Analysis

Data analysis was analyzed with GraphPad Prism software version 8. Data were expressed with mean ± standard error of mean (SEM). Differences between groups were analyzed using repeated-measure analysis of variance or t-test. A p value of less than 0.05 is considered statistically significant.

## Data Availability Statement

The original contributions presented in the study are included in the article/supplementary material. Further inquiries can be directed to the corresponding authors.

## Author Contributions

Conception and design of the study: GH, ZW, RW. Acquisition, analysis, and interpretation of data: LT, GL. Contribution of administrative, experimental, analytic, or material support: YZ, CH, YG, ZG, YH, RM, YL, BS. Writing—original draft preparation: LT, ZW. Editing: GH. All authors contributed to the article and approved the submitted version.

## Funding

This work was supported by the National Natural Sciences Foundation of China (grant nos. 81971473, 81771684, 82171753) and the Beijing Natural Sciences Foundation (grant no. 7192145).

## Conflict of Interest

The authors declare that the research was conducted in the absence of any commercial or financial relationships that could be construed as a potential conflict of interest.

## Publisher’s Note

All claims expressed in this article are solely those of the authors and do not necessarily represent those of their affiliated organizations, or those of the publisher, the editors and the reviewers. Any product that may be evaluated in this article, or claim that may be made by its manufacturer, is not guaranteed or endorsed by the publisher.

## References

[1] Han G Chen G Shen B Li Y. Tim-3: An Activation Marker and Activation Limiter of Innate Immune Cells. Front Immunol (2013) 4:449. doi: 10.3389/fimmu.2013.00449 24339828 PMC3857553

[2] Wang Z Chen J Wang M Zhang L Yu L. One Stone, Two Birds: The Roles of Tim-3 in Acute Myeloid Leukemia. Front Immunol (2021) 12:618710. doi: 10.3389/fimmu.2021.618710 33868234 PMC8047468

[3] Ocana-Guzman R Torre-Bouscoulet L Sada-Ovalle I. TIM-3 Regulates Distinct Functions in Macrophages. Front Immunol (2016) 7:229. doi: 10.3389/fimmu.2016.00229 27379093 PMC4904032

[4] Mohammadzadeh A Rad IA Ahmadi-Salmasi B. CTLA-4, PD-1 and TIM-3 Expression Predominantly Downregulated in MS Patients. J Neuroimmunol (2018) 323:105–8. doi: 10.1016/j.jneuroim.2018.08.004 30196822

[5] Afshar B Khalifehzadeh-Esfahani Z Seyfizadeh N Rezaei Danbaran G Hemmatzadeh M Mohammadi H. The Role of Immune Regulatory Molecules in Multiple Sclerosis. J Neuroimmunol (2019) 337:577061. doi: 10.1016/j.jneuroim.2019.577061 31520791

[6] Zhang R Li H Bai L Duan J. Association Between T-Cell Immunoglobulin and Mucin Domain 3 (TIM-3) Genetic Polymorphisms and Susceptibility to Autoimmune Diseases. Immunol Invest (2019) 48(6):563–76. doi: 10.1080/08820139.2019.1599009 31044630

[7] Wang Z Li G Dou S Zhang Y Liu Y Zhang J. Tim-3 Promotes Listeria Monocytogenes Immune Evasion by Suppressing Major Histocompatibility Complex Class I. J Infect Dis (2020) 221(5):830–40. doi: 10.1093/infdis/jiz512 31586389

[8] Unanue ER Turk V Neefjes J. Variations in MHC Class II Antigen Processing and Presentation in Health and Disease. Annu Rev Immunol (2016) 34:265–97. doi: 10.1146/annurev-immunol-041015-055420 26907214

[9] Yau AC Piehl F Olsson T Holmdahl R. Effects of C2ta Genetic Polymorphisms on MHC Class II Expression and Autoimmune Diseases. Immunology (2017) 150(4):408–17. doi: 10.1111/imm.12692 PMC5343355 27861821

[10] DeNardo DG Ruffell B. Macrophages as Regulators of Tumour Immunity and Immunotherapy. Nat Rev Immunol (2019) 19(6):369–82. doi: 10.1038/s41577-019-0127-6 PMC7339861 30718830

[11] International Multiple Sclerosis Genetics C Wellcome Trust Case Control C Sawcer S Hellenthal G Pirinen M Spencer CC. Genetic Risk and a Primary Role for Cell-Mediated Immune Mechanisms in Multiple Sclerosis. Nature (2011) 476(7359):214–9. doi: 10.1038/nature10251 PMC3182531 21833088

[12] Kaskow BJ Baecher-Allan C. Effector T Cells in Multiple Sclerosis. Cold Spring Harb Perspect Med (2018) 8(4):a029025. doi: 10.1101/cshperspect.a029025 29358315 PMC5880159

[13] Masternak K Muhlethaler-Mottet A Villard J Zufferey M Steimle V Reith W. CIITA Is a Transcriptional Coactivator That Is Recruited to MHC Class II Promoters by Multiple Synergistic Interactions With an Enhanceosome Complex. Genes Dev (2000) 14(9):1156–66. doi: 10.1101/gad.14.9.1156 PMC316580 10809673

[14] Badawi AH Kiptoo P Siahaan TJ. Immune Tolerance Induction Against Experimental Autoimmune Encephalomyelitis (EAE) Using A New PLP-B7AP Conjugate That Simultaneously Targets B7/CD28 Costimulatory Signal and TCR/MHC-II Signal. J Mult Scler (Foster City) (2015) 2(1):1000131. doi: 10.4172/2376-0389.1000131 26140285 PMC4484621

[15] Schmitz K Wilken-Schmitz A Vasic V Brunkhorst R Schmidt M Tegeder I. Progranulin Deficiency Confers Resistance to Autoimmune Encephalomyelitis in Mice. Cell Mol Immunol (2020) 17(10):1077–91. doi: 10.1038/s41423-019-0274-5 PMC7609649 31467413

[16] Wang Z Sun D Chen G Li G Dou S Wang R. Tim-3 Inhibits Macrophage Control of Listeria Monocytogenes by Inhibiting Nrf2. Sci Rep (2017) 7:42095. doi: 10.1038/srep42095 28205579 PMC5311873

[17] Barreiro LB Tailleux L Pai AA Gicquel B Marioni JC Gilad Y. Deciphering the Genetic Architecture of Variation in the Immune Response to Mycobacterium Tuberculosis Infection. Proc Natl Acad Sci USA (2012) 109(4):1204–9. doi: 10.1073/pnas.1115761109 PMC3268270 22233810

[18] Miller S Tsou PS Coit P Gensterblum-Miller E Renauer P Rohraff DM. Hypomethylation of STAT1 and HLA-DRB1 Is Associated With Type-I Interferon-Dependent HLA-DRB1 Expression in Lupus CD8+ T Cells. Ann Rheum Dis (2019) 78(4):519–28. doi: 10.1136/annrheumdis-2018-214323 PMC6679955 30674474

[19] Coste C Gerard N Dinh CP Bruguiere A Rouger C Leong ST. Targeting MHC Regulation Using Polycyclic Polyprenylated Acylphloroglucinols Isolated From Garcinia Bancana. Biomolecules (2020) 10(9):1266. doi: 10.3390/biom10091266 PMC7563419 32887413

[20] Liang J Wang L Wang C Shen J Su B Marisetty AL. Verteporfin Inhibits PD-L1 Through Autophagy and the STAT1-IRF1-TRIM28 Signaling Axis, Exerting Antitumor Efficacy. Cancer Immunol Res (2020) 8(7):952–65. doi: 10.1158/2326-6066.CIR-19-0159 PMC8204534 32265228

[21] Li G Tang L Hou C Wang Z Gao Y Dou S. Peripheral Injection of Tim-3 Antibody Attenuates VSV Encephalitis by Enhancing MHC-I Presentation. Front Immunol (2021) 12:667478. doi: 10.3389/fimmu.2021.667478 34025669 PMC8138436

[22] Fernando MM Stevens CR Walsh EC De Jager PL Goyette P Plenge RM. Defining the Role of the MHC in Autoimmunity: A Review and Pooled Analysis. PLoS Genet (2008) 4(4):e1000024. doi: 10.1371/journal.pgen.1000024 18437207 PMC2291482

[23] Tintore M Vidal-Jordana A Sastre-Garriga J. Treatment of Multiple Sclerosis - Success From Bench to Bedside. Nat Rev Neurol (2019) 15(1):53–8. doi: 10.1038/s41582-018-0082-z 30315270

[24] Faissner S Plemel JR Gold R Yong VW. Progressive Multiple Sclerosis: From Pathophysiology to Therapeutic Strategies. Nat Rev Drug Discov (2019) 18(12):905–22. doi: 10.1038/s41573-019-0035-2 31399729

[25] Stys PK Zamponi GW van Minnen J Geurts JJ. Will the Real Multiple Sclerosis Please Stand Up? Nat Rev Neurosci (2012) 13(7):507–14. doi: 10.1038/nrn3275 22714021

[26] Lodygin D Hermann M Schweingruber N Flugel-Koch C Watanabe T Schlosser C. Publisher Correction: Beta-Synuclein-Reactive T Cells Induce Autoimmune CNS Grey Matter Degeneration. Nature (2019) 567(7749):E15. doi: 10.1038/s41586-019-1047-0 30867589

[27] Androdias G Reynolds R Chanal M Ritleng C Confavreux C Nataf S. Meningeal T Cells Associate With Diffuse Axonal Loss in Multiple Sclerosis Spinal Cords. Ann Neurol (2010) 68(4):465–76. doi: 10.1002/ana.22054 20687208

[28] Giralt M Molinero A Hidalgo J. Active Induction of Experimental Autoimmune Encephalomyelitis (EAE) With MOG35-55 in the Mouse. Methods Mol Biol (2018) 1791:227–32. doi: 10.1007/978-1-4939-7862-5_17 30006713

[29] Glatigny S Bettelli E. Experimental Autoimmune Encephalomyelitis (EAE) as Animal Models of Multiple Sclerosis (MS). Cold Spring Harb Perspect Med (2018) 8(11):a028977. doi: 10.1101/cshperspect.a028977 29311122 PMC6211376

[30] Enders U Lobb R Pepinsky RB Hartung HP Toyka KV Gold R. The Role of the Very Late Antigen-4 and its Counterligand Vascular Cell Adhesion Molecule-1 in the Pathogenesis of Experimental Autoimmune Neuritis of the Lewis Rat. Brain (1998) 121( Pt 7):1257–66. doi: 10.1093/brain/121.7.1257 9679778

[31] Wilmes AT Reinehr S Kuhn S Pedreiturria X Petrikowski L Faissner S. Laquinimod Protects the Optic Nerve and Retina in an Experimental Autoimmune Encephalomyelitis Model. J Neuroinflamm (2018) 15(1):183. doi: 10.1186/s12974-018-1208-3 PMC6002998 29903027

[32] Xiao Y Chen J Wang J Guan W Wang M Zhang L. Acute Myeloid Leukemia Epigenetic Immune Escape From Nature Killer Cells by ICAM-1. Front Oncol (2021) 11:751834. doi: 10.3389/fonc.2021.751834 34722306 PMC8548470

[33] Wang Z Guan W Wang M Chen J Zhang L Xiao Y. AML1-ETO Inhibits Acute Myeloid Leukemia Immune Escape by CD48. Leuk Lymphoma (2021) 62(4):937–43. doi: 10.1080/10428194.2020.1849680 33225787

